# Exogenous Cytokinins Increase Grain Yield of Winter Wheat Cultivars by Improving Stay-Green Characteristics under Heat Stress

**DOI:** 10.1371/journal.pone.0155437

**Published:** 2016-05-20

**Authors:** Dongqing Yang, Yong Li, Yuhua Shi, Zhengyong Cui, Yongli Luo, Mengjing Zheng, Jin Chen, Yanxia Li, Yanping Yin, Zhenlin Wang

**Affiliations:** 1 State Key Laboratory of Crop Biology, Agronomy College of Shandong Agricultural University, Tai’an, Shandong, P. R. China; 2 Agricultural Bureau of Yanzhou District, Jining, Shandong, P. R. China; Murdoch University, AUSTRALIA

## Abstract

Stay-green, a key trait of wheat, can not only increase the yield of wheat but also its resistance to heat stress during active photosynthesis. Cytokinins are the most potent general coordinator between the stay-green trait and senescence. The objectives of the present study were to identify and assess the effects of cytokinins on the photosynthetic organ and heat resistance in wheat. Two winter wheat cultivars, Wennong 6 (a stay-green cultivar) and Jimai 20 (a control cultivar), were subjected to heat stress treatment from 1 to 5 days after anthesis (DAA). The two cultivars were sprayed daily with 10 mg L^-1^ of 6-benzylaminopurine (6-BA) between 1 and 3 DAA under ambient and elevated temperature conditions. We found that the heat stress significantly decreased the number of kernels per spike and the grain yield (*P* < 0.05). Heat stress also decreased the zeatin riboside (ZR) content, but increased the gibberellin (GA_3_), indole-3-acetic acid (IAA), and abscisic acid (ABA) contents at 3 to 15 DAA. Application of 6-BA significantly (*P* < 0.05) increased the grain-filling rate, endosperm cell division rate, endosperm cell number, and 1,000-grain weight under heated condition. 6-BA application increased ZR and IAA contents at 3 to 28 DAA, but decreased GA_3_ and ABA contents. The contents of ZR, ABA, and IAA in kernels were positively and significantly correlated with the grain-filling rate (*P* < 0.05), whereas GA_3_ was counter-productive at 3 to 15 DAA. These results suggest that the decrease in grain yield under heat stress was due to a lower ZR content and a higher GA_3_ content compared to that at elevated temperature during the early development of the kernels, which resulted in less kernel number and lower grain-filling rate. The results also provide essential information for further utilization of the cytokinin substances in the cultivation of heat-resistant wheat.

## Introduction

Global increase in ambient temperature is a critical factor affecting plant growth [1]. High temperature stress is one of the primary abiotic constraints to wheat (*Triticum aestivum* L.) production in many countries [2, 3], especially in the Huang-Huai-Hai plain of China, during the period from late May to early June, at around the time of anthesis and grain-filling [1, 4–6]. Heat stress causes many biochemical and physiological changes at the cellular and whole plant level that affect crop yield [7, 8]. The photosynthetic apparatus in leaves are impaired by heat stress due to damages to the ultra-structure of organelles and reduction in RuBisco activity [9–11]; grain filling in wheat is, therefore, affected by heat stress due to the declined activity and duration of leaf photosynthesis [12–14]. In addition, high temperature during the grain-filling period also markedly affects the carbon (C) metabolism, like the synthesis of starch [15]. The decreased activities of key enzymes for starch synthesis were observed to be largely responsible for the reduction in starch accumulation in wheat grains under heat stress [16].

Plant hormones, such as cytokinins, play a key role in the stimulation of cell division, nucleic acid metabolism, and root-shoot interactions, particularly under stress [17–20]. Application of cytokinin could retard leaf senescence and improve creeping bent grass tolerance to heat stress by increasing the antioxidant activities and decreasing the lipid peroxidation [21]. Treatment of broccoli florets with 6-benzylaminopurine (6-BA) reduced the chlorophyllase levels and inhibited chlorophyll degradation [22]. It was concluded that higher zeatin (Z) and zeatin riboside (ZR) levels in the grains increased grain filling percentage during the early and middle grain-filling stages [23, 24]. In addition, cytokinins in grains could mediate cell division in rice endosperm at early grain filling stages, and therefore regulate the sink size of the grain [25].

It is also documented that cytokinins could retain the levels of chlorophyll in the photosynthetic apparatus in barley, wheat and maize [26–28]. In addition, some mutants, such as stay-green mutants of wheat, are characterized by longer green leaf duration and delayed senescence [29]. This can be the result of alterations in hormone metabolism and signaling, particularly affecting networks involving cytokinins and ethylene [30]. However, our understanding of differences between stay-green and non-stay-green varieties in yield-forming mechanisms under heat stress, including for example differences in endosperm cell division and hormonal alterations, is very limited. Moreover, knowledge of the effects of cytokinin application on grain filling in different stay-green cultivars under heat stress is scant. In the present study, we conducted two experiments, with two different stay-green trait wheat cultivars, by spraying exogenous 6-BA (or adding ZR) under ambient and elevated temperature conditions. We attempted to (i) identify the effect of heat stress on endosperm cell division, grain filling, and endogenous hormonal alterations; (ii) elucidate the effect of exogenous cytokinins on endosperm cell division and grain filling in stay-green wheat under heat stress; and (iii) improve our understanding on the regulating mechanism of cytokinins during yield formation in stay-green winter wheat under heat stress.

## Materials and Methods

### Plant Materials and Growth Condition

Experiments were carried out during the growing seasons of 2011–2013 (the field experiments were conducted in 2011–2012 and the indoor experiments in 2012–2013) at Shandong Agricultural University Farm, Tai’an, Shandong province, China (36°09′N, 117°09′E, 128 m above sea level). Plants of two winter wheat (*Triticum aestivum* L.) cultivars, Wennong 6 (a stay-green cultivar) and Jimai 20 (a non-stay-green cultivar), were grown in experimental plots. Plot size was 7.5 m^2^ (3 m × 2.5 m) with 10 rows (0.25 m between rows). The soil contained 12.3 g kg^–1^ organic matter, 0.91 g kg^–1^ total N, 0.0872 g mg kg^–1^ available N, 0.0086 g kg^–1^ Olsen-P, and 0.0575 g kg^–1^ Olsen-K. Fertilizer, at the rate of 120 kg ha^-1^ N, 100 kg ha^-1^ P_2_O_5_, and 100 kg ha^-1^ K_2_O, was applied as basal fertilizer before planting, with another 120 kg ha^-1^ N applied as topdressing at stage 31 in the scale of Zadoks et al. [31]. Seeds were sown on October 10, 2011 and October 10, 2012 at a density of 225 plants m^–2^. Pests, diseases, and weeds were controlled by appropriate chemical applications during the growing period. Other cultural practices followed the precision high-yielding cultivation system of Yu [32].

### Treatments and Experimental Design

#### Field experiments

**Fig 1:**
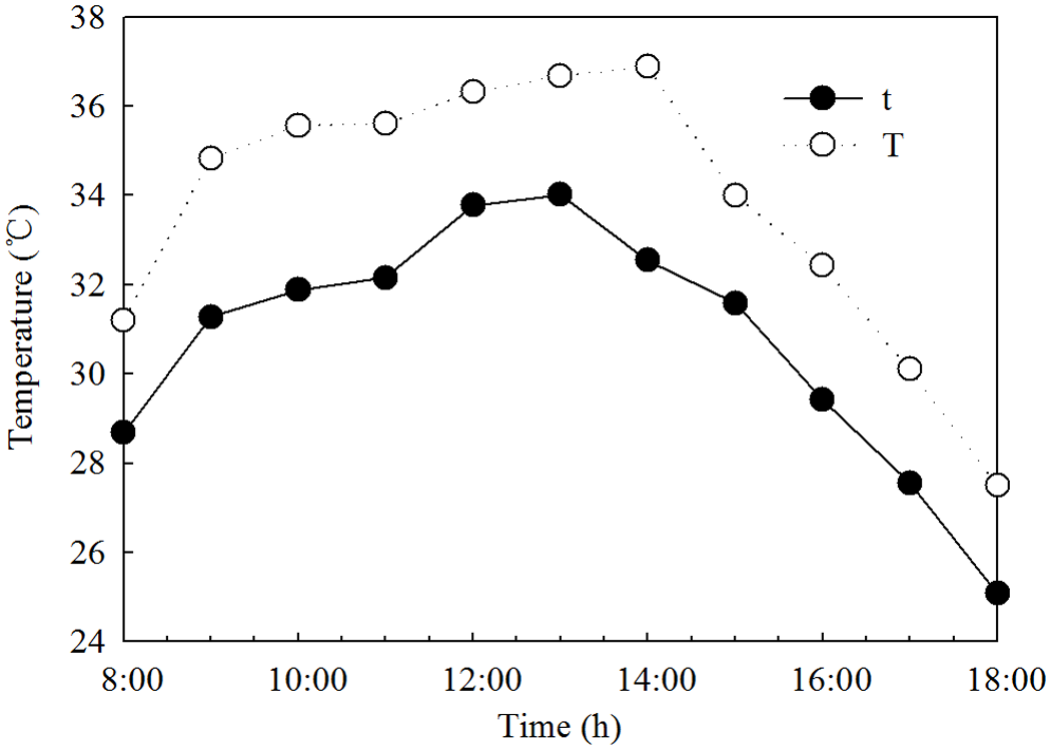
Diurnal changes of mean temperature inside and outside the sheds. T and t represent the elevated temperature and ambient temperature conditions, respectively. Hourly value was averaged over 5 days’ measurements.

The field experiment was a 2 × 2 × 2 [two cultivars (Wennong 6 and Jimai 20), two levels of air temperature (ambient and elevated temperature), and two exogenous regulators treatment (water and 6-BA)] factorial design with 8 treatments. CC and 6-BA treatments represent spraying with water and 6-BA under ambient temperature condition, respectively. HC and H6-BA treatment represent spraying with water and 6-BA under elevated temperature condition, respectively. Each of the treatments had three plots as repetitions in a completely randomized block design. The elevated temperature treatment was conducted on 6 May 2012 for 5 days, from 1 to 5 days after anthesis (DAA). The method for providing heat stress was modified from that described by Xu et al. [33]. Transparent plastic sheds (3 m × 3 m × 1.5 m, light transmittance of 92%) were set in the treatment plots of the two varieties from 08:00 to 18:00 hours. The sheds were left in place at night. The sheds had open air shafts (0.2 m × 0.2 m) on all sides to keep them ventilated and make the same concentrations of carbon dioxide and air humidity on the inside and out. No shading was set in the ambient temperature condition. Temperatures inside and outside the sheds were measured by a self-recording instrument, ZDR-20 (Hangzhou Zeda Instruments Co., Ltd). Fig 1 shows hourly average values of temperatures inside and outside the sheds. Plants were sprayed with 0.01 g L^-1^ 6-BA at a rate of 100 L ha^-1^ starting 1 to 3 DAA at 5:00 P.M. Water was sprayed in the control treatment. All the solutions contained Tween-20, as a surfactant, at a final concentration of 0.5% (v/v). Each treatment was administered in an area of 7.5 m^2^ with three replications.

#### Indoor experiments

The experiments had 3 factors, each with two levels: factor 1: ZR; factor 2: high temperature for 2 days; factor 3: cultivar. Uniform spikes flowering on the same day (7 May 2013) were tagged for each cultivar. One day after anthesis, 120 tagged spikes with flag leaves, from each cultivar were cut at the penultimate stem for use *in vitro* culture. The different cultures were divided into the following four treatment groups: (1) spikes cultured without ZR at 25/20°C (day/night) for 10 days (NZR); (2) spikes cultured with ZR at 25/20°C for 10 days (ZR); (3) spikes cultured without ZR at 35/20°C for 2 days and then for 8 days at 25/20°C (HNZR); (4) spikes cultured with ZR at 35/20°C for 2 days and then for 8 days at 25/20°C (HZR). The concentrations of sucrose and L-glutamine (nitrogen source) in the culture medium were 120 and 30 mM, respectively. Other nutrients concentrations in the culture medium were as described by Xie et al. [34]. The culture medium without ZR was considered as control. ZR was dissolved in nutrient solution at a concentration of 0.1 mM. Ten detached ears were sterilized with sodium hypochlorite solution (1% available chlorine) and then cut to 10 cm below the flag leaf node under sterile distilled water, inserted into a sterile conical flask (150 mL) containing 90 mL of sterile liquid culture medium for each treatment; each treatment was carried out in triplicates. The spikes were incubated in a growth cabinet (Intelligent Light Incubator GXZ-500D, Ningbo, China) under a day/night temperature cycle of 25/20°C (35/20°C for heat stress), 16-h photoperiod (8 h darkness), and a photosynthetic active radiation of 400 μmol m^-2^ s^-1^. The culture medium and conical flasks were replaced daily during the culture period. After 10 days of culture, the grains were separated and used for counting kernel number per spike and kernel weight.

#### Sampling

From 3 DAA, thirty spikes from each plot in the field experiments were sampled at 3-days intervals until 35 DAA. Spikes were divided into two parts, one stored at -40°C for endogenous hormone measurement and the other dehulled for endosperm cell number measurement. The middle spikelets (from sixth to sixteenth) of two cultivars were detached for the assay in this study. The first and second basal kernels on each spikelet were detached and considered as the superior kernels. The third and fourth distal kernels were the inferior kernels. Another thirty spikes were sampled at 3-days intervals according to the experiment, dried at 70°C to constant weight, dehulled, and weighed. These data were used to simulate the grain-filling process. Both the grain filling process and endosperm cell division process were fitted by the Richards’s growth equation, as described by Yang et al. [35]:

$$W(M)=\frac{A}{{\left(1+Be^{-kt}\right)}^{\frac{1}{N}}}$$ (1)

where, W was the grain weight (g), M was the endosperm cell number, A was the final grain weight (g) or the maximum endosperm cell number, t was the time after anthesis (d), and B, k, and N were the coefficients determined by regression. The active grain-filling (or the active period of endosperm cell division) period (D) was defined as the period during which W (or M) constituted from 5% (t_1_) to 95% (t_2_) of A.

Grain filling rate (G) or endosperm division rate (R) was calculated as the derivative of Eq (1):

$$G(R)=\frac{AKBe^{-kt}}{N{\left(1+Be^{-kt}\right)}^{\frac{N+1}{N}}}$$ (2)

Integration of the Eq (2) gave the mean grain-filling rate [the mean endosperm cell division rate (R_mean_)]: $G_{mean}(R_{mean})=\frac{Ak}{2(N+2)}$, and the maximum grain-filling rate [the maximum endosperm cell division rate (R_max_)]: $G_{\text{max}}(R_{\text{max}})=Ak{\left(1+N\right)}^{\frac{-(N+1)}{N}}$.

At maturity, the plants in an area (no heads were sampled from this area) of 1 m^2^ were harvested to determine the yield, number of kernels per spike, and 1,000-grain weight; each measurement was performed on plants from three different plots.

#### Assays for endosperm cell number

The method for isolation, counting, and calculation of the endosperm cell number per endosperm was according to Yang et al. [35]. Ten superior or inferior kernels were fixed in FAA solution (formalin:acetic acid:ethyl alcohol (70%), 1:1:8, v/v) for 48 h. Embryos were removed from these kernels, and were stained with hematoxylin solution for 24 h, washed several times with distilled water, and then hydrolyzed in 0.4% (w/v) cellulose at 40°C for 8 h and oscillated. Isolated endosperm cells were diluted to 10 mL. One milliliter solution was filtered through 0.45 μm hydrophobic membrane, and a sample was placed on a glass slide with a drop of glycerol. Using a light microscope, the endosperm cell number was counted in ten fields of view for each membrane.

#### Determination of endogenous hormones

The method for extraction and purification of ZR, gibberellin (GA_3_), indole-3-acetic acid (IAA), and abscisic acid (ABA) was modified from that described by Zhao et al. [36]. A sample of 1 g of kernels from each treatment was ground to a powder in liquid nitrogen and 4 mL acetonitrile extraction medium with 30 mg sodium diethyldithiocarbamate added as an antioxidant. The extract was incubated at 4°C for 12 h and centrifuged at 5,000 × *g* for 15 min. The residue was further extracted twice with the same solvent. The supernatant was concentrated to a residue under low pressure at 37°C by rotary evaporation and redissolved in 8 mL 0.4 mol L^–1^ Na-phosphate buffer (pH 8.0), followed by addition of 6 mL chloroform and oscillation to remove the pigment. Insoluble polyvinylpyrrolidone (0.15 g) was added to the aqueous phase, and the mixture was centrifuged at 10,000 × *g* for 10 min, followed by removal of 5 mL supernatant, which was adjusted to pH 3.0 with pure formic acid. The aqueous phase was extracted twice with 3 mL ethyl acetate. The ethyl acetate phase was concentrated by rotary evaporation under low pressure and redissolved in 1 mL mobile phase (acetonitrile: methanol: 0.6% acetic acid, 5:50:45, v/v). Finally, the hormone extract was filtered through 0.2 μm hydrophobic membranes, and 10-μL samples were injected into a Waters Symmetry C18 column (4.6 mm × 150.0 mm, 5 μm) using the mobile phase. The flow rate was held at 0.6 mL min^–1^ and the peaks were detected with a photodiode array detector (Waters 2998 Separation Module, USA) by determining the absorbance at 254 nm.

#### Statistical analysis

Data in all the experiments were analyzed by PASW software version 18.0 (IBM Corp., Chicago, IL, USA). Multi-factor repeated measure analysis of variance was used to test significant differences at *P* < 0.05 among individual genotypes, heat stress treatments, and hormone treatments.

## Results

### Grain Yield and Grain-Filling Process

Analysis of kernel weight, kernel number, active grain filling period and yield made it possible to identify the effect of application of 6-BA and the 6-BA × heat stress interaction (Tables 1 and 2). Compared with the ambient temperature treatment, all the yield composition elements including kernel weight, grain yield, and kernel number, in both the cultivars, significantly declined at the elevated temperature (Table 1). Kernel weight and grain yield in Wennong 6 were significantly (*P* < 0.05) decreased by 0.9 g and 19.65%, respectively. In contrast, these values in Jimai 20 were decreased by 0.9 g and 26.22%, respectively. Superior and inferior kernels number per spike in Wennong 6 were decreased by 3.7 and 8.2 kernels∙spike^-1^, respectively. In contrast, these values in Jimai 20 were decreased by 1.3 and 4.4 kernels∙spike^-1^, respectively. In spite of the fact that the decrease in kernels number of Wennong 6 was more than that of Jimai 20, Wennong 6 had the higher grain yield at the elevated temperature. This is because both kernel number and kernel weight of Wennong 6 were higher than those of Jimai 20 at the elevated temperature. (Table 1). Application of 6-BA could significantly (*P* < 0.05) increase both the kernel weight and yield in the two cultivars. Exogenous 6-BA significantly (*P* < 0.05) increased the inferior kernel number in Wennong 6 (0.7 kernels spike^-1^), and significantly (*P* < 0.05) increased superior and inferior kernel number in Jimai 20 (1.4 and 2.1 kernels spike^-1^) in the elevated temperature treatment. The weight of superior kernels was markedly (*P* < 0.05) greater than that of inferior kernels in the two cultivars under both the ambient and elevated temperature conditions. Moreover, analyzing kernel weight, kernel number and yield in both cultivars at the elevated temperature with exogenous 6-BA application, the results suggested that the effects of exogenous cytokinin on yield components in Jimai 20 at the elevated temperature were more effective than that in Wennong 6 (Table 1).

**Table 1:** Effect of exogenous 6-BA on factors of wheat yield under ambient (AT) and elevated temperature (ET) conditions.

Cultivar	Treatment		Kernel number per spike			Kernel weight (g 1000 kernels^-1^)	Grain yield (g m^-2^)
			Total mount	Superior	Inferior		
Wennong 6	AT	CC	45.8 a	31.6 a	14.2 a	39.7 b	724.8 b
		6-BA	45.4 a	31.1 a	14.3 a	40.8 a	773.9 a
	ET	HC	33.9 b	27.9 b	6.0 c	38.8 c	582.4 f
		H6-BA	34.8 b	28.1 b	6.7 b	39.4 b	638.1 e
Jimai 20	AT	CC	34.1 b	26.8 c	7.3 bc	32.4 e	652.2 d
		6-BA	34.9 b	27.0 c	7.9 b	33.4 d	667.2 c
	ET	HC	28.4 d	25.5 d	2.9 d	31.5 f	481.2 g
		H6-BA	31.9 c	26.9 c	5.0 c	33.5 d	559.8 f
*P*-value		*P*(H)	0.0001	0.0001	0.0001	0.0001	0.0001
		*P* (G)	0.0001	0.0001	0.0001	0.0001	0.0001
		*P* (R)	0.0231	0.0413	0.0001	0.0001	0.0001
		*P* (H×G)	0.0001	0.0001	0.0001	0.0027	0.9978
		*P* (H×R)	0.0342	0.0036	0.0123	0.2529	0.0002
		*P* (G×R)	0.2646	0.3362	0.3402	0.0374	0.7998
		*P* (H×G×R)	0.0919	0.4645	0.4928	0.0005	0.2217

*P* values (*P*) for the effects of temperature (H), cultivars (G), 6-BA (R) and their interactions (H × G, H × R, G × R, H × G × R) are shown. Values followed by different letters within the columns are significantly different at the 0.05 probability level.

**Table 2:** Effect of exogenous 6-BA on active grain filling period and mean grain filling rate of superior and inferior kernels under ambient (AT) and elevated temperature (ET) conditions.

Cultivar	Treatment		Active grain filling period (d)		Mean grain filling rate (mg per kernel d^-1^)	
			Superior	Inferior	Superior	Inferior
Wennong 6	AT	CC	30.59 cd	28.55 a	1.50 a	1.33 b
		6-BA	34.28 ab	28.53 a	1.47 b	1.41 a
	ET	HC	32.54 bc	26.70 b	1.36 cd	1.29 c
		H6-BA	35.56 a	28.96 a	1.37 c	1.30 c
Jimai 20	AT	CC	27.80 e	23.81 c	1.26 f	1.08 e
		6-BA	27.46 e	24.63 c	1.34 d	1.17 d
	ET	HC	28.70 de	22.92 d	1.17 g	1.05 f
		H6-BA	29.54 de	22.74 d	1.30 e	1.10 e
*P*-value		*P*(H)	0.0075	0.0001	0.0001	0.0001
		*P* (G)	0.0001	0.0001	0.0001	0.0001
		*P* (R)	0.0026	0.0021	0.0001	0.0001
		*P* (H×G)	0.9035	0.1026	0.0003	0.0497
		*P* (H×R)	0.8047	0.1227	0.0015	0.0003
		*P* (G×R)	0.0075	0.0585	0.0001	0.0497
		*P* (H×G×R)	0.3754	0.0007	0.6768	0.2209

*P* values (*P*) for the effects of temperature (H), cultivars (G), 6-BA (R) and their interactions (H × G, H × R, G × R, H × G × R) are shown. Values followed by different letters within the columns are significantly different at the 0.05 probability level.

**Fig 2:**
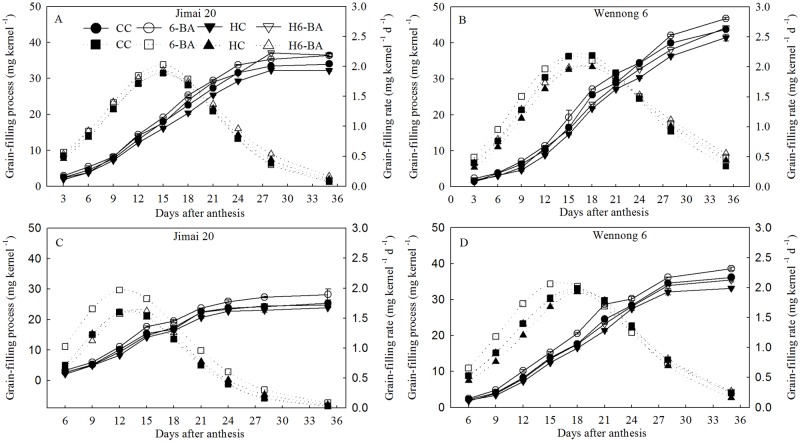
Grain weight and grain-filling rate for superior (A, B) and inferior kernels (C, D). CC and 6-BA represent treatments with water and 6-BA under non-heated condition, respectively. HC and H6-BA represent treatment with water and 6-BA under heated condition, respectively. Solid line and dotted line represent endosperm grain weight and grain-filling rate, respectively. Vertical bars represent ± standard error of the mean (n = 3) where they exceed the size of the symbol.

Nevertheless, elevated temperature prolonged the active grain-filling period only for superior kernels; the mean grain-filling rate was significantly decreased for both the cultivars, irrespective of the superior or inferior kernels (Table 2). Spraying 6-BA increased the mean grain-filling rate of superior and inferior kernels in two cultivars. These results indicate that the improved kernels weight of 6-BA-treated Wennong 6 and Jimai 20 were due to increase of grain-filling duration and the increase of grain-filling rate, respectively. As expected, the grain-filling rate in all the treatments showed a parabolic change (Fig 2). Application of 6-BA increased the grain weight and grain-filling rate at all the grain filling stages under both ambient and elevated temperature conditions.

**Fig 3:**
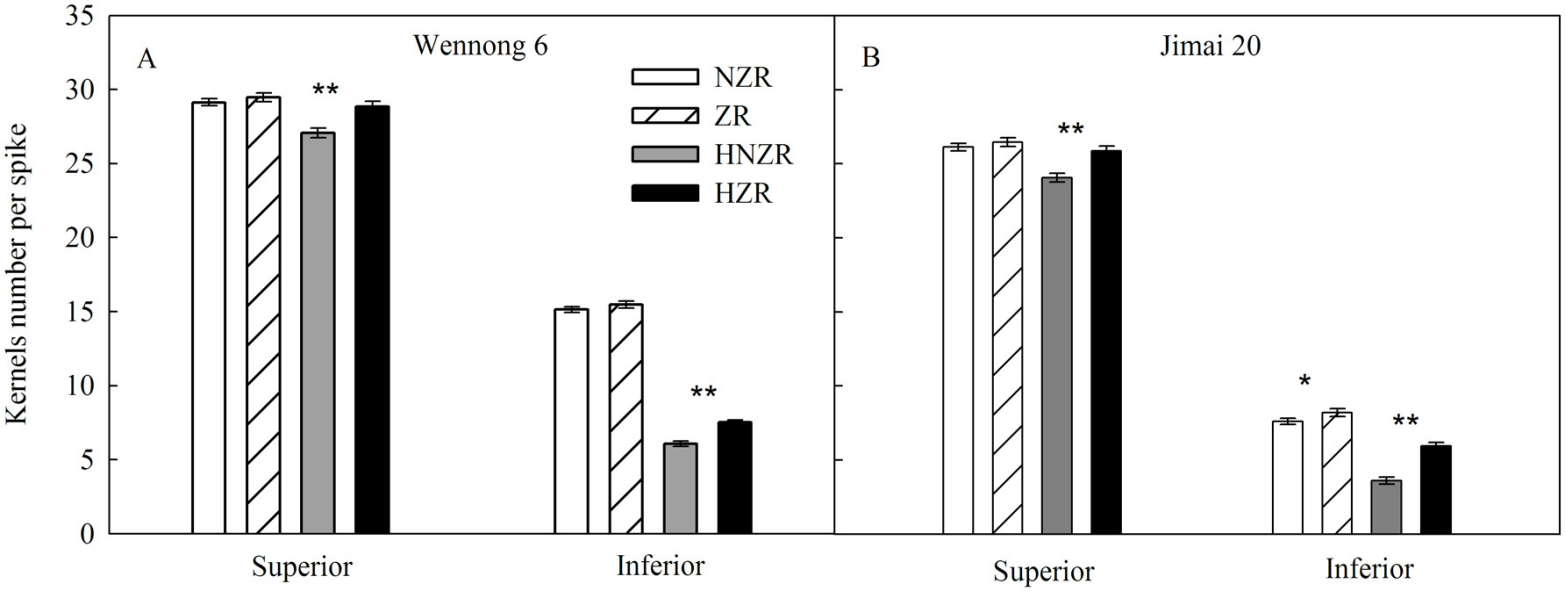
Effects of high temperature and exogenous ZR on the kernel number at 10 days after anthesis. NZR and ZR represent detached spike culture without and with ZR under 25/20°C condition, respectively. HNZR and HZR represent detached spike culture without and with ZR under 35/20°C condition, respectively. Vertical bars represent ± standard error of the mean (n = 9) where they exceed the size of the symbol. *, ** denote significant differences at *P* < 0.05, *P* < 0.01, respectively.

**Fig 4:**
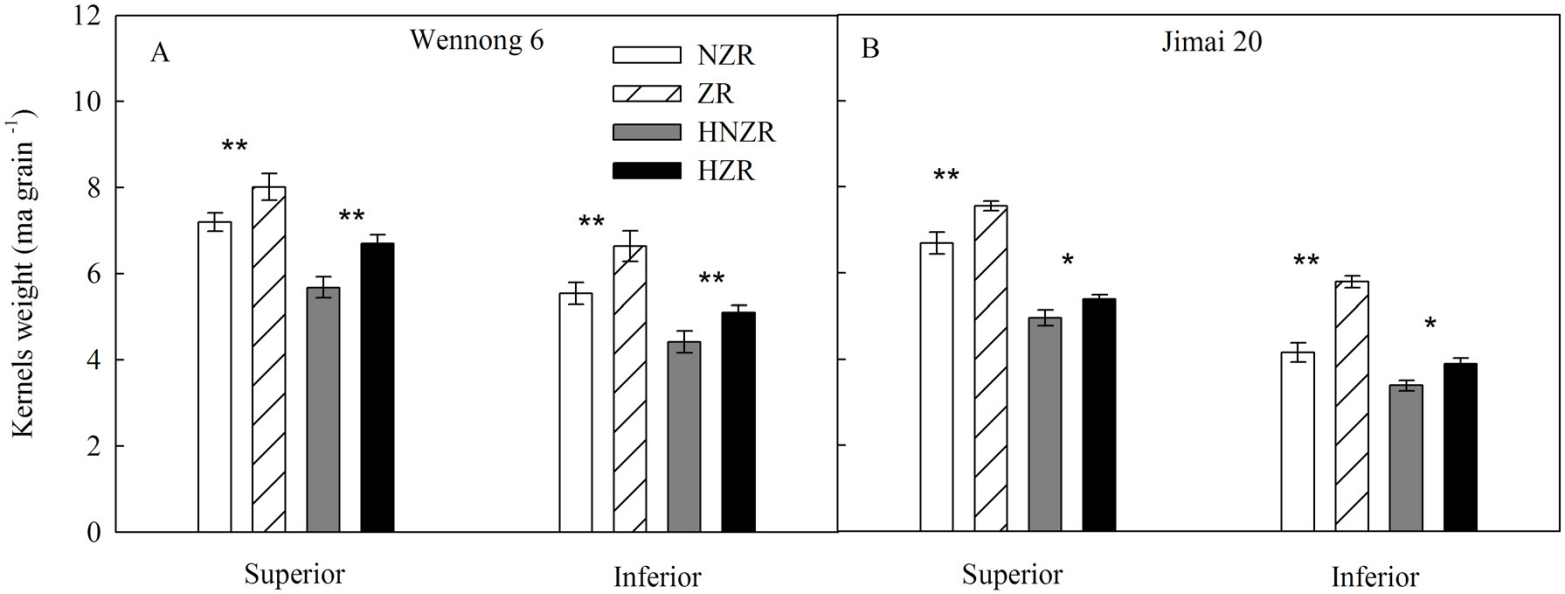
Effects of high temperature and exogenous ZR on kernels weight at 10 days after anthesis. NZR and ZR represent detached spike culture without and with ZR under 25/20°C condition, respectively. HNZR and HZR represent detached spike culture without and with ZR under 35/20°C condition, respectively. Vertical bars represent ± standard error of the mean (n = 9) where they exceed the size of the symbol. *, ** denote significant difference at *P* < 0.05, *P* < 0.01, respectively.

In the indoor experiments, highly significant temperature × cultivar and temperature × 6-BA interactions (*P* < 0.01) were observed for kernel weight. Kernel weight and inferior kernels number per spike in the two cultivars were significantly (*P* < 0.05) decreased when the spikes were cultured under 35/20°C condition (Fig 3). Kernel weight was significantly increased with the addition of ZR in culture medium under both 25/20°C and 35/20°C conditions (Fig 4). Exogenous ZR increased the inferior kernel number per spike in the two cultivars under 35/20°C condition. These results show that manipulating ZR supply can affect kernel number and weight under heat stress.

### Endosperm Cell Number and Endosperm Cell Division Parameters

**Fig 5:**
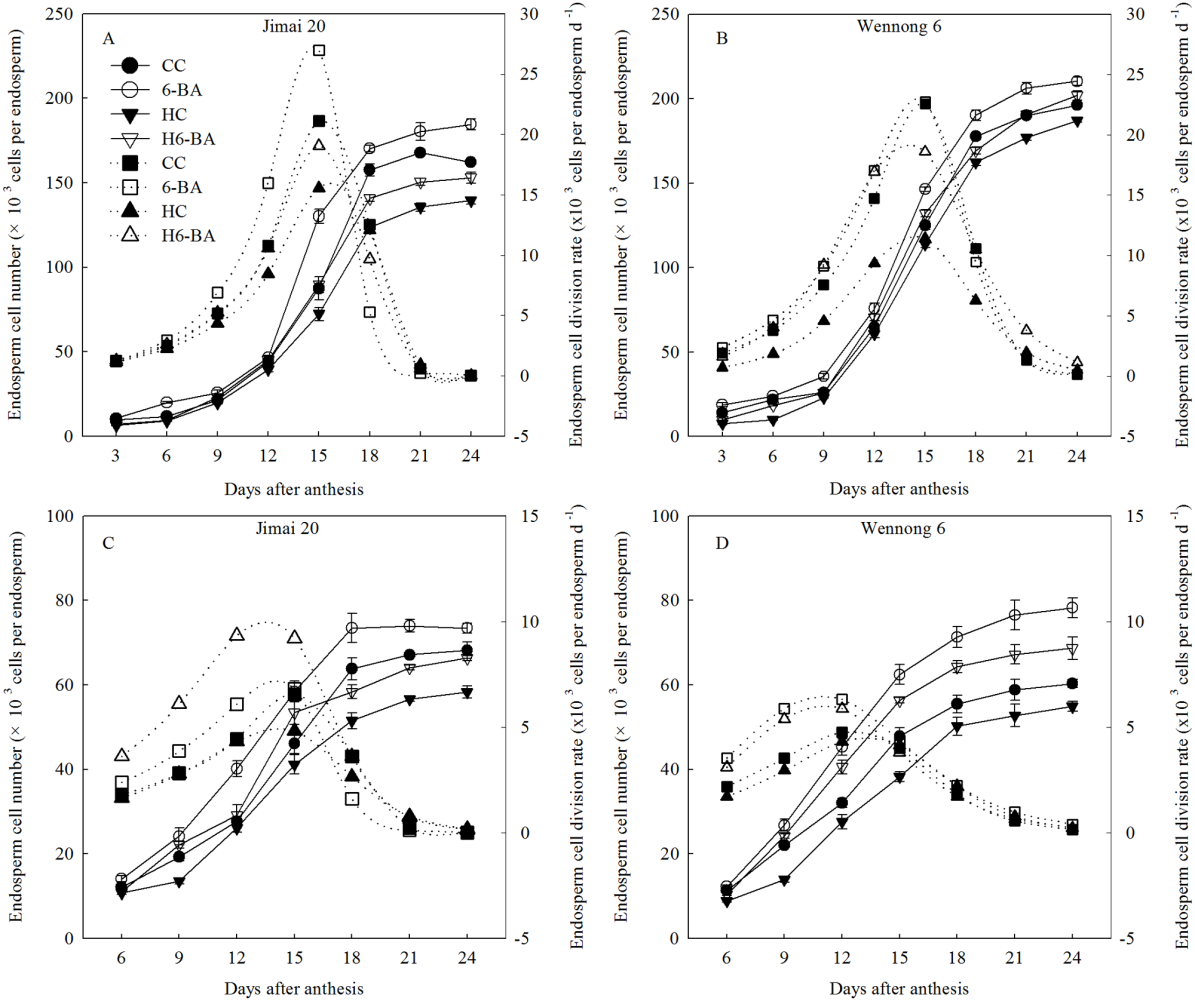
Endosperm cell number and endosperm cell division rate for superior (A, B) and inferior kernels (C, D). CC and 6-BA represent treatment with water and 6-BA under non-heated condition, respectively. HC and H6-BA represent treatment with water and 6-BA under heated condition, respectively. Solid line and dotted line represent endosperm cell number and endosperm cell division rate, respectively. Vertical bars represent ± standard error of the mean (n = 9) where they exceed the size of the symbol.

Mean endosperm cell division rate and max endosperm cell number of superior and inferior kernels was also reduced (Table 3). As expected, the endosperm cell division rates in all the treatments showed a parabolic change. Both endosperm cell number and endosperm cell division rate were significantly (*P* < 0.05) influenced by heat stress and 6-BA at all the endosperm cell division stages, where heat stress inhibited the endosperm cell division and reduced the cell division rates in both the cultivars from 3 to 18 DAA (Fig 5). There were highly significant temperature × cultivar interactions (*P* < 0.01) for the mean endosperm cell division rate of superior grains. When spraying with exogenous 6-BA, endosperm cell number and endosperm cell division rates in the two cultivars were significantly (*P* < 0.05) increased under both the ambient and elevated temperature conditions.

**Table 3:** Effect of exogenous 6-BA on the mean endosperm cell division rate and max endosperm cell number of superior and inferior kernels under ambient (AT) and elevated temperature (ET) conditions.

Cultivar	Treatment		Max endosperm cell number (× 10^3^ cells per endosperm)		Mean endosperm cell division rate (× 10^3^ cells per endosperm d^-1^)	
			Superior	Inferior	Superior	Inferior
Wennong 6	AT	CC	194.00 c	72.85 bc	14.22 b	3.09 d
		6-BA	208.54 a	79.54 a	13.33 c	4.17 b
	ET	HC	187.10 d	59.40 e	12.87 c	2.94 d
		H6-BA	201.92 b	69.35 cd	13.33 c	4.07 b
Jimai 20	AT	CC	166.51 f	67.88 d	14.42 b	4.04 b
		6-BA	180.69 e	74.18 b	16.63 a	4.62 a
	ET	HC	138.24 h	58.37 e	10.87 d	3.16 cd
		H6-BA	152.11 g	65.06 d	10.96 d	3.53 c
*P*-value		*P*(H)	0.0001	0.0030	0.0001	0.0001
		*P* (G)	0.0001	0.0001	0.1114	0.01
		*P* (R)	0.0001	0.0001	0.0023	0.0001
		*P* (H×G)	0.0001	0.2797	0.0001	0.0003
		*P* (H×R)	0.9947	0.4269	0.1554	0.6709
		*P* (G×R)	0.8278	0.4269	0.0001	0.0036
		*P* (H×G×R)	0.9212	0.5306	0.0001	0.4920

*P* values (*P*) for the effects of temperature (H), cultivars (G), 6-BA (R) and their interactions (H × G, H × R, G × R, H × G × R) are shown. Values followed by different letters within columns are significantly different at the 0.05 probability level.

### Endogenous Hormone Levels in Kernels

**Fig 6:**
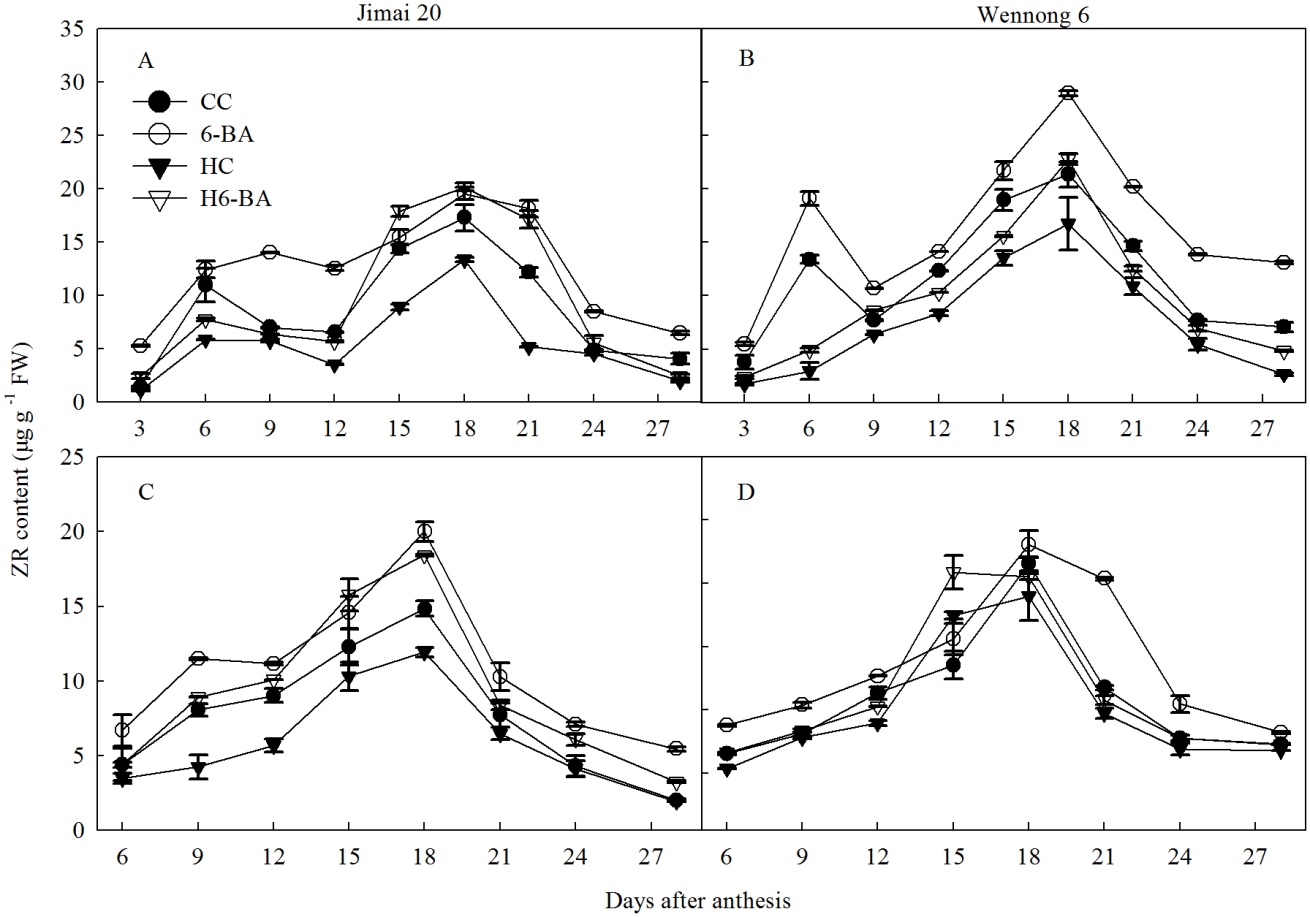
ZR content for superior (A, B) and inferior kernels (C, D). CC and 6-BA represent treatment with water and 6-BA under non-heated condition, respectively. HC and H6-BA represent treatment with water and 6-BA under heated condition, respectively. Vertical bars represent ± standard error of the mean (n = 3) where they exceed the size of the symbol.

Fig 6 shows that ZR levels in superior and inferior kernels in both the cultivars increased during 3 to 18 DAA, reached their highest level at 18 DAA, and then decreased sharply with grain filling. Compared with CC treatment, HC treatment significantly (*P* < 0.05) decreased the ZR levels in superior and inferior kernels in both the cultivars at all grain filling stages. Both 6-BA and H6-BA treatments markedly increased the ZR content in the kernels of both the cultivars from 3 to 28 DAA, compared with CC and HC treatment, respectively.

**Fig 7:**
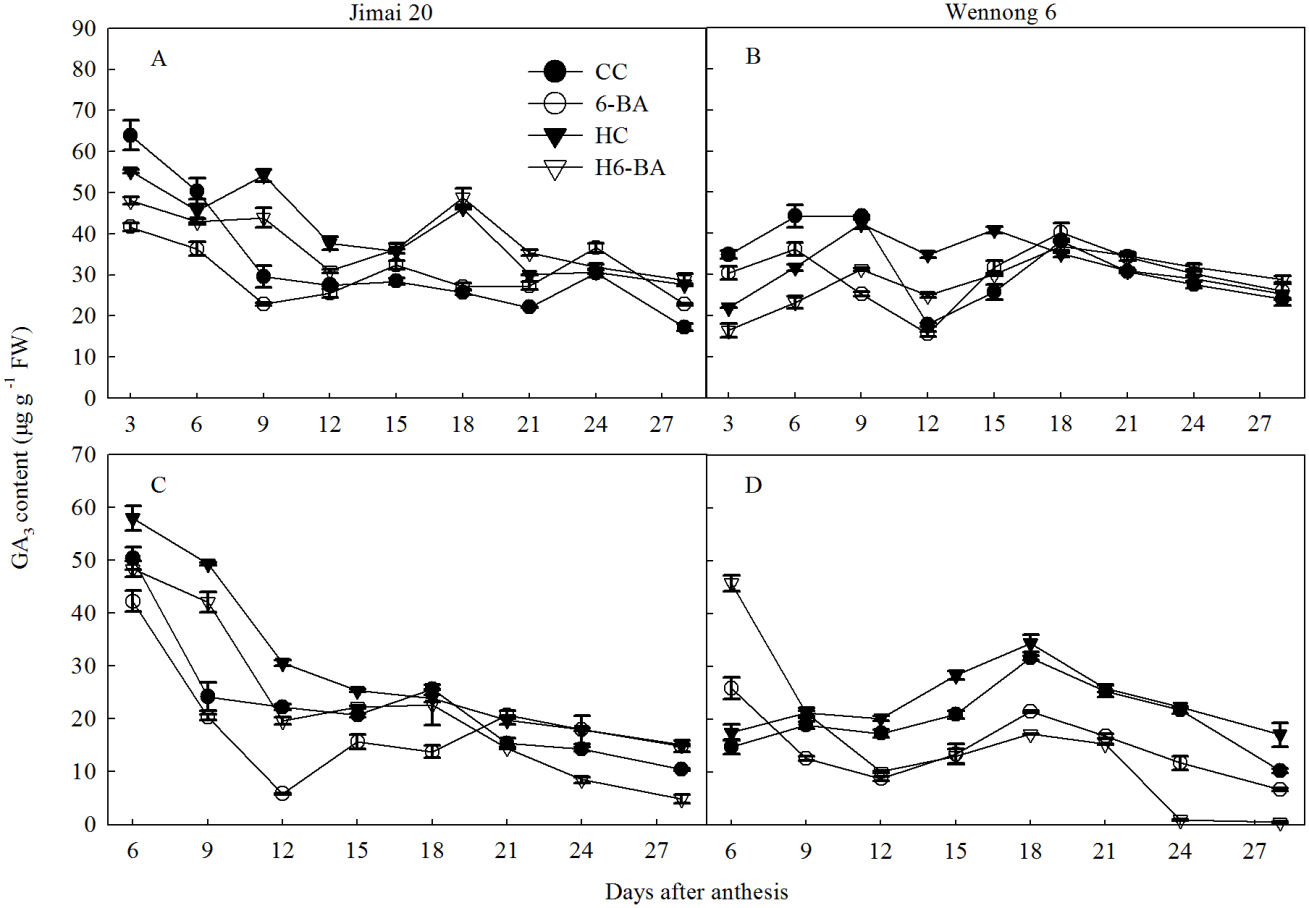
GA_3_ content in superior (A, B) and inferior kernels (C, D). CC and 6-BA represent treatment with water and 6-BA under non-heated condition, respectively. HC and H6-BA represent treatment with water and 6-BA under heated condition, respectively. Vertical bars represent ± standard error of the mean (n = 3) where they exceed the size of the symbol.

GA_3_ content in both the kernels were high initially at the early grain filling stage (Fig 7) and declined up to 28 DAA. Compared with CC treatment, HC treatment decreased GA_3_ content from 3 to 6 DAA in the superior kernels in both the cultivars, but increased GA_3_ content from 9 to 28 DAA in the superior kernels of Jimai 20. In contrast, from 6 to 28 DAA, the GA_3_ content in the inferior kernels of the two cultivars was significantly (*P* < 0.05) increased by heat stress. Under control temperature condition, 6-BA application decreased GA_3_ content from 3 to 12 DAA in superior and 6 to 18 DAA in the inferior kernels of Jimai 20. However, 6-BA application increased the GA_3_ content from 15 to 28 DAA in superior and 21 to 28 DAA in inferior kernels of Jimai 20. Compared with HC treatment, H6-BA treatment decreased the GA_3_ content from 3 to 12 DAA in superior and 9 to 28 DAA in inferior kernels of the two cultivars.

**Fig 8:**
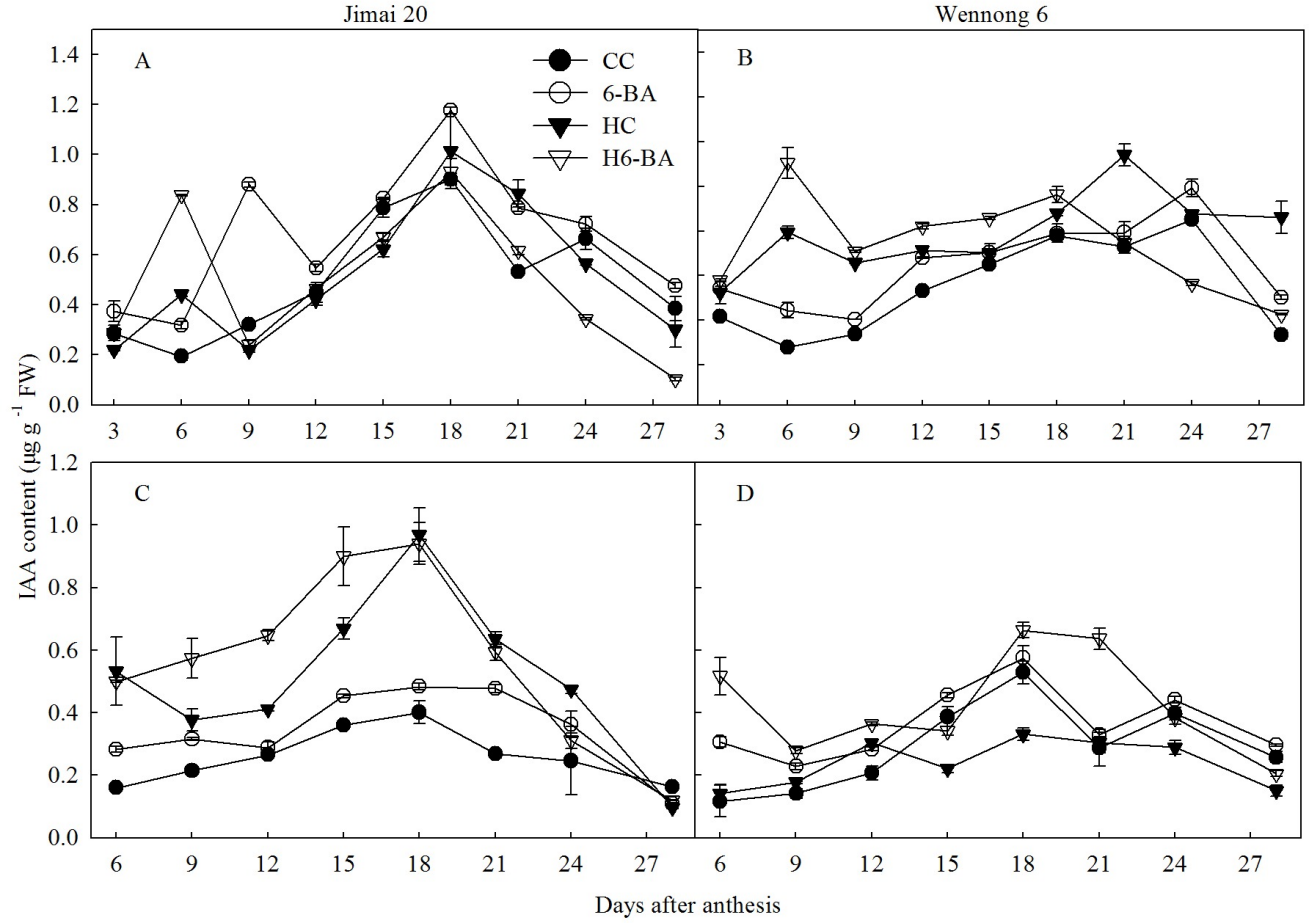
IAA content in superior (A, B) and inferior kernels (C, D). CC and 6-BA represent treatment with water and 6-BA under non-heated condition, respectively. HC and H6-BA represent treatment with water and 6-BA under heated condition, respectively. Vertical bars represent ± standard error of the mean (n = 3) where they exceed the size of the symbol.

IAA content first increased and then decreased, reaching maximum values at 18 DAA for superior and inferior kernels (Fig 8). Under all the treatments, the peak values in IAA concentration occurred at 21 and 18 DAA for superior and inferior kernels in Wennong 6. Application of 6-BA markedly increased the IAA content of two cultivars at all the grain filling stages. HC treatment decreased the IAA content from 9 to 15 DAA in the superior kernels, but increased it from 6 to 24 DAA in the inferior kernels of Jimai 20. Compared with the HC treatment, the H6-BA treatment increased the IAA content from 3 to 18 DAA in the superior and 6 to 28 DAA in the inferior kernels of Wennong 6.

**Fig 9:**
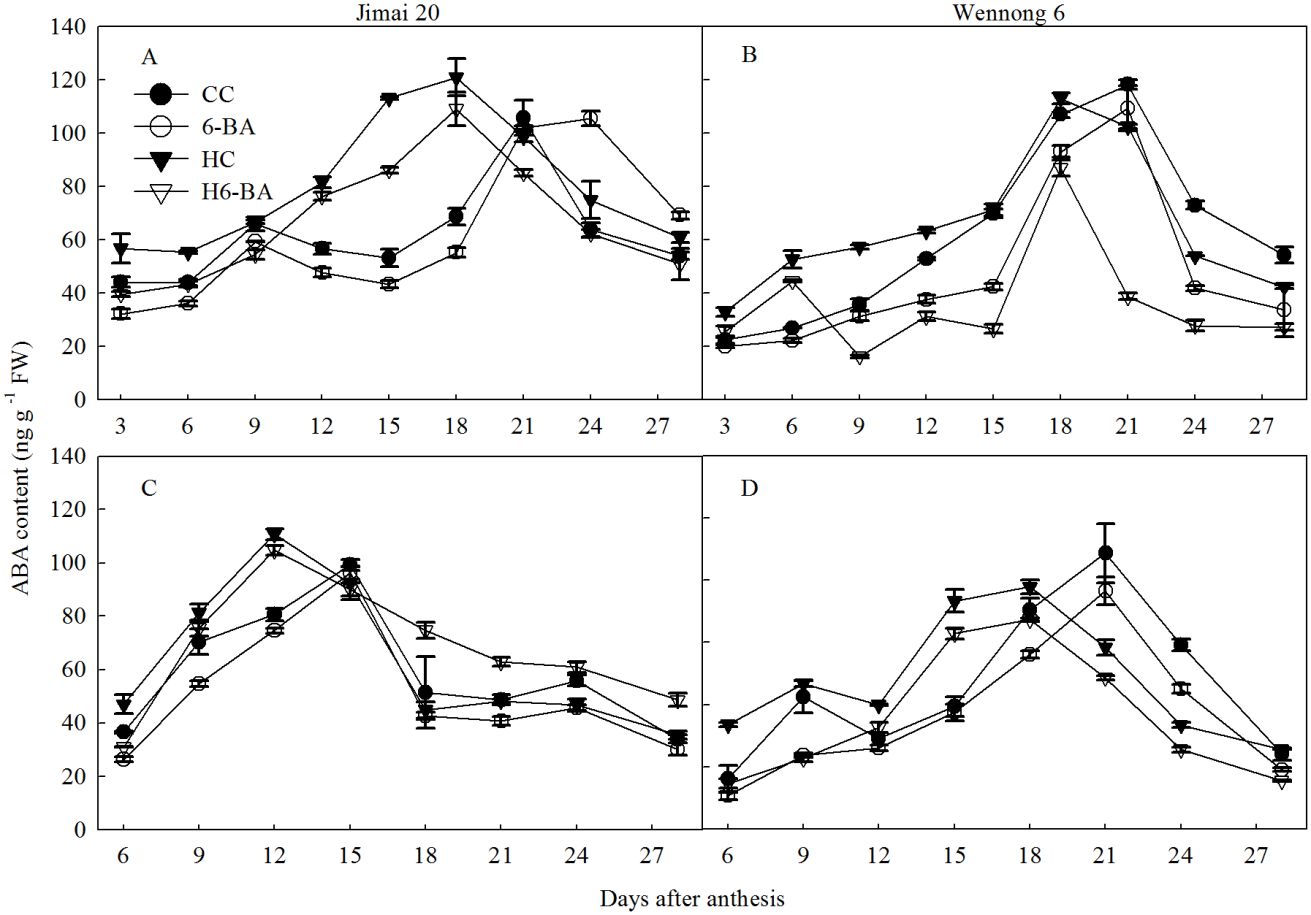
ABA content in superior (A, B) and inferior kernels (C, D). CC and 6-BA represent treatment with water and 6-BA under non-heated condition, respectively. HC and H6-BA represent treatment with water and 6-BA under heated condition, respectively. Vertical bars represent ± standard error of the mean (n = 3) where they exceed the size of the symbol.

Fig 9 displays the ABA content in the two cultivars. ABA content in the kernels sharply increased during the early grain filling stage, reached a maximum at 18 DAA for superior and at 12 DAA for inferior kernels of Jimai 20, and dropped very quickly, thereafter. Application of 6-BA decreased the endogenous ABA contents in the two types of kernels in the two cultivars under both control temperature and heat stress conditions.

### Relationships between Hormone Contents in Kernels and Grain-Filling Rate

**Fig 10:**
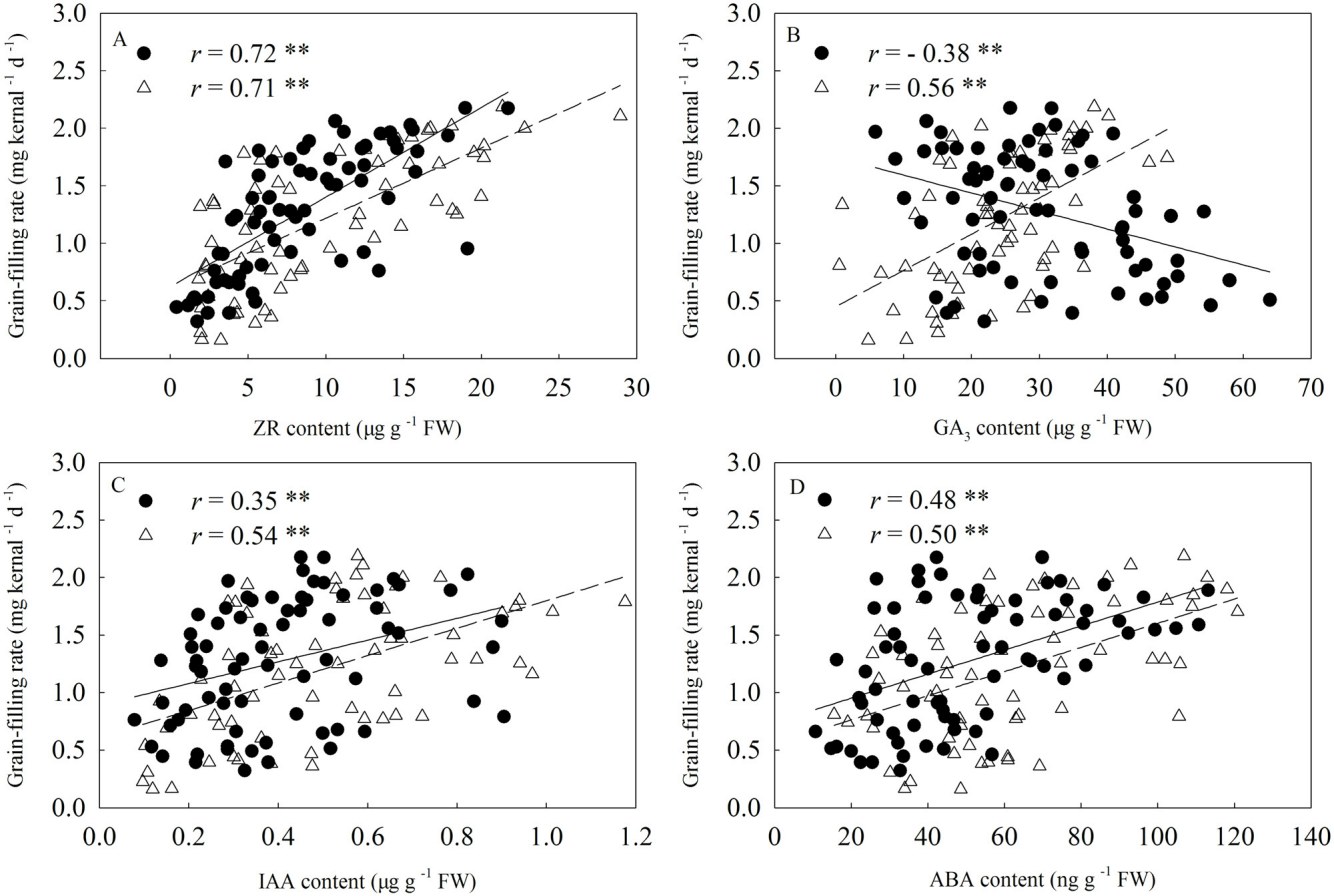
Relationship between grain-filling rate and the hormone concentrations in kernels. Closed circles and open triangles represent hormone concentrations at 3 to 15 days after anthesis (DAA) and at 18 to 28 DAA, respectively. The solid and short dotted line represent the regression line between grain-filling rate and hormone concentrations at 3 to 15 DAA (n = 72) and at 18 to 28 DAA (n = 64), respectively. Correlation coefficients (*r*) are calculated and asterisks (**) represent significance at the 0.01 probability level.

Significant correlation was observed between the hormone content and grain-filling rate (Fig 10). The ZR content of kernels positively and significantly (*r* = 0.72 **, 0.71 **, *P* < 0.01) correlated with the grain-filling rate at 3 to 15 DAA and at 18 to 28 DAA, respectively. However, GA_3_ content in the kernels negatively and significantly (*r* = − 0.38 **, *P* < 0.01) correlated with the grain-filling rate at 3 to 15 DAA, whereas it significantly and positively (*r* = 0.56 **, *P* < 0.01) correlated with the grain-filling rate at 18 to 28 DAA. IAA contents in kernels significantly and positively (*r* = 0.35 **, 0.54**, respectively.) correlated with the grain-filling rate. Furthermore, ABA concentration significantly and positively correlated with the grain-filling rate (*r* = 0.48 **, 0.50 **, *P* < 0.01).

### Relationships between Hormone Contents in Kernels and Endosperm Cell Division Rate

**Fig 11:**
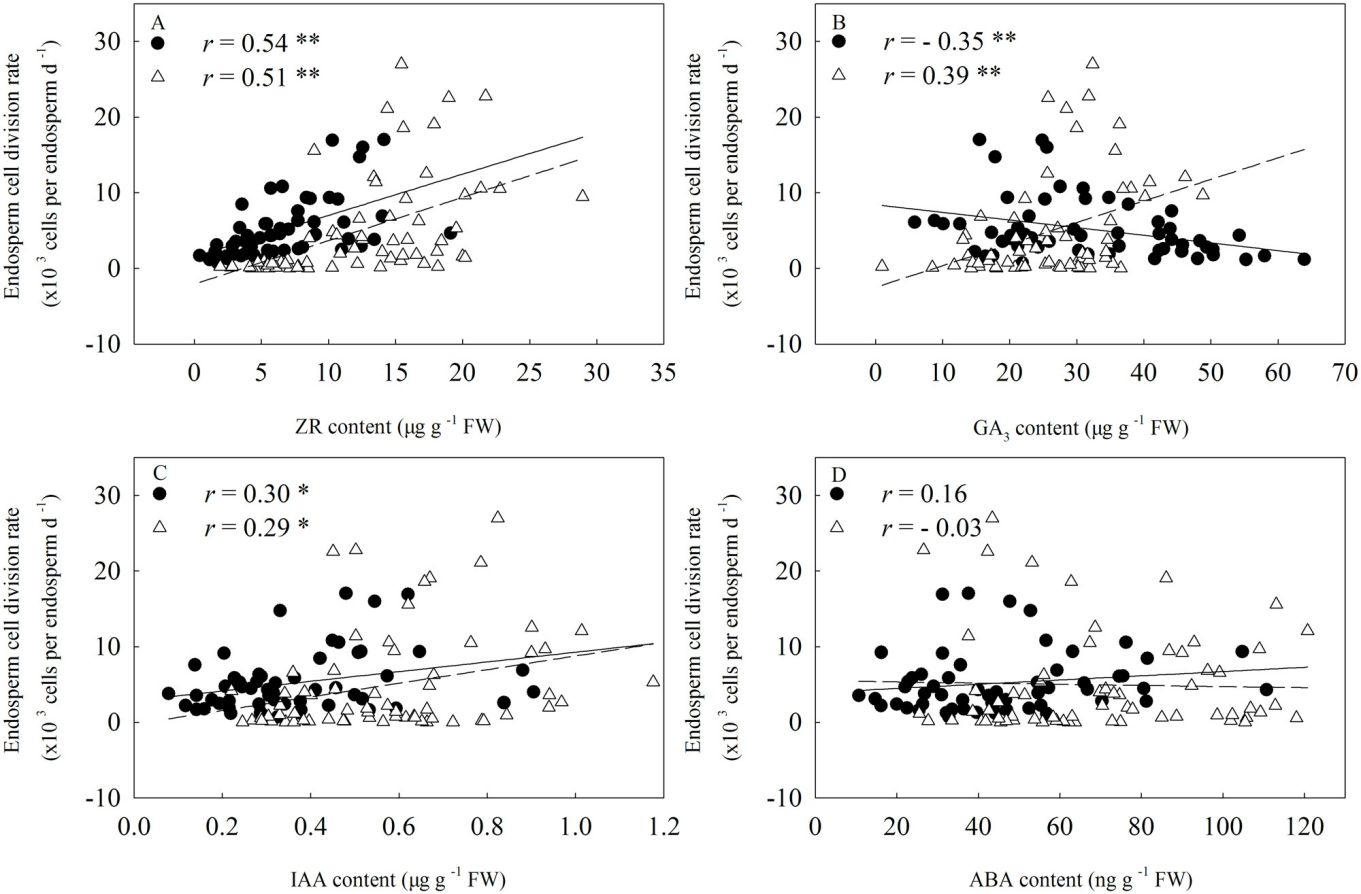
Relationship between endosperm cell division rate and hormone concentrations in the kernels. Closed circles and open triangles represent hormone concentration at 3 to 12 days after anthesis (DAA) and at 15 to 24 DAA, respectively. The solid and short dotted line represent the regression line between endosperm cell division rate and hormone concentration at 3 to 15 DAA (n = 56) and at 18 to 28 DAA (n = 64), respectively. Correlation coefficients (*r*) are calculated and asterisks (**) represent significance at the 0.01 probability level and asterisks (*) represent significance at the 0.05 probability level.

ZR content in the kernels positively and significantly (*r* = 0.54* and 0.51**) correlated with the endosperm cell division rate at 3 to 12 DAA and at 15 to 24 DAA, respectively (Fig 11A). The GA_3_ content in kernels negatively (*r* = − 0.35 **, *P* < 0.01) correlated with the endosperm cell division rate at 3 to 12 DAA, but this relationship was significantly positive at 15 to 24 DAA (Fig 11B). IAA content in the kernels positively correlated with the rate of endosperm cell division at all the stages (Fig 11C). No significant response of ABA content in the kernels to the rate of endosperm cell division at all the stages were detected (Fig 11D).

## Discussion

Wheat is very sensitive to high temperature and needs an optimum temperature for pollination and grain production [6, 37]. Short period (3 to 5 days) of high temperature (>35°C) stress post-anthesis could reduce the grain yield by decreasing the starch content and dry matter accumulation in grain [38–41]. The effect of short periods of exposure to high temperatures (>31°C) on wheat grain yields are thought to be equivalent to a 2–3°C increase in the seasonal mean temperature [14]. In the present work, we set the maximum and mean temperature of heat stress treatment, which reached 37°C and 33°C, respectively (Fig 1). The results showed that grain yield of the two cultivars were significantly (*P* < 0.05) decreased by elevated temperature treatments (Table 1). The heat stress treatment reduced the number of kernels per spike and the kernel weight. We also found that the number of endosperm cells in the superior and inferior kernels of the two cultivars was significantly (*P* < 0.05) reduced by the heat stress, whereas the inferior kernels proved to be a bigger disadvantage in the response to the adverse environment. Although the active grain-filling period was a little prolonged under heat stress, especially for the superior kernels, it was just the wheat adaptation that had to be compensated by the decreased sink capacity stress. The grain yield of Wennong 6 was higher than that in Jimai 20 under both heated and non-heated conditions. The grain yield of Wennong 6 was reduced by 19.65%. In contrast, the values in Jimai 20 was reduced by 26.22%. The results indicate that non-staygreen cultivar is susceptible to heat stress. Analyzes its reason, it mainly is that endosperm cell number of Wennong 6 is insusceptible to heat stress. The grain-filling rate and endosperm cell division rate of Wennong 6 generally remain high under elevated temperature treatment, compared to the ambient temperature treatment. Stay-green cultivar could matain the supply of assimilated carbon to grain during the grain-filling period at the elevated temperature, which was in accordance with the conclusion that heat tolerance can be improved by selecting and developing wheat genotypes [42]. It is possible that endogenous hormones are involved in regulating kernels development.

Plant endogenous hormones play important roles in determining sink strength and seed weight during development of the caryopsis and are involved in the plant’s response to stresses [25, 43]. Hays et al. [3] stated that 10 days after pollination, heat stress induced abortion of kernels owing to a rapid increase in ethylene production in the developing kernels. Zeatin could promote endosperm cell division and increased the sink capacity, resulting in more assimilate accumulation [44]. In this experiment, the data analysis demonstrated that the effect of exogenous 6-BA on yield composition in Jimai 20 at the elevated temperature was greater than that in stay-green cultivar Wennong 6. Conversely, exogenous 6-BA enhanced active superior grain filling period, mean inferior endosperm cell division rate and max endosperm cell number of Wennong 6 much more than that of Jimai 20 at the elevated temperature (Tables 1–3). It was obvious that great difference was existed between Wennong 6 and Jimai 20 in response to 6-BA under the elevated temperature treatment. This may be due to stay-green cultivar and non-stay-green cultivar had different cytokinin metabolism, which was consistent with recent findings [45]. Banowetz et al. [19] found that high temperature reduced the endogenous cytokinin content resulting in the reduction of kernel weight, and the number of kernels, whereas it could be reversed by supplementing with exogenous cytokinin or IAA [46]. In this study, heat stress decreased endogenous ZR content, but increased GA_3_ content at 3 to 15 DAA. Exogenous 6-BA resulted in a decrease in the endogenous GA_3_ content at early grain filling stages and maintained a higher endogenous ZR content from 3 to 28 DAA under heat stress in both the cultivars. The results indicated that ZR, IAA, and ABA content showed significant and positive correlation with the grain-filling rate and endosperm cell division rate. However, GA_3_ content negatively correlated with the grain-filling rate and endosperm cell division rate at the early grain filling stages, whereas the correlation was positive at the late grain filling stages, partly consistent with the observations of Xu et al. [47]. Exogenous 6-BA significantly prolonged active grain filling period in both cultivars (Table 2) and enhanced ZR content (Fig 6) under heat stress. This indicated that exogenous cytokinins could change cytokinin metabolism [45], sustain longer active photosynthetic period during the grain filling stage, transfer more assimilates to the grain [48], improve the resistance to abiotic stresses post anthesis [29], and finally increase yield under heat stress [30]. The above traits were referred to as the stay green trait in crops by other researchers.

## Conclusion

Based on our results and those reported in previous studies, we can summarize the mechanism of grain yield reduction under heat stress as follows: heat stress (i) decreased the grain sink strength by reducing the rate of endosperm cell division and the number of endosperm cells owing to the decrease in ZR content and the increase in GA_3_ content at the early endosperm cell division stages and (ii) affected the grain carbohydrate accumulation by decreasing the grain-filling rate. In our study, the decline in the endosperm cell division rate and grain-filling rate were mitigated by exogenous cytokinins, especially under heat stress treatment. Therefore, alleviation of heat stress in wheat grain yield by exogenous cytokinins could be related to expanding grain sink strength by increasing the endosperm cell number and accelerating grain carbohydrate accumulation by accelerating the grain filling. All the processes were performed keeping the ZR and IAA content at high levels during the grain-filling period.

## Supporting Information

S1 Dataset Dataset of Figs. Diurnal changes of mean temperature inside and outside the sheds, grain weight, endosperm cell number, endogenous ZR, GA3, IAA, ABA content for superior and inferior kernels under ambient (AT) and elevated temperature (ET) conditions, and relationship between grain-filling rate, endosperm cell division rate and the hormone concentrations in kernels. Figs 1–11 were plotted based on this excel file.
(XLS)

S2 Dataset Dataset of Tables. Yield components, grain filling period, grain filling rate, the mean endosperm cell division rate and max endosperm cell number of superior and inferior kernels under ambient (AT) and elevated temperature (ET) conditions. Tables 1–3 were created based on this excel file.
(XLS)

S1 Fig The phenotype of Jimai 20 and Wennong 6 at 30 days after anthesis. (TIF)
