# Supplementary figures and images for: Exogenous Cytokinins Increase Grain Yield of Winter Wheat Cultivars by Improving Stay-Green Characteristics under Heat Stress
Source: PLoS One. 2016 May 20;11(5):e0155437. doi: 10.1371/journal.pone.0155437 (PMC4874672; doi:10.1371/journal.pone.0155437)

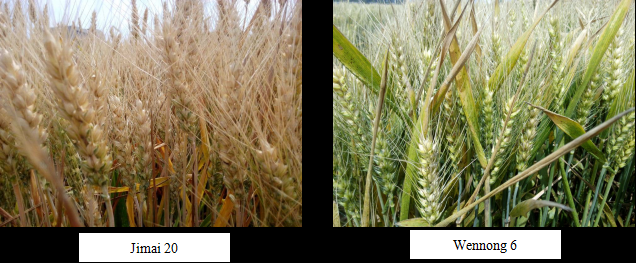

Supplement: S1 Fig — (TIF) [file pone.0155437.s003.tif]
